# Enhanced skin delivery of biomacromolecules using hollow sponge spicules

**DOI:** 10.1002/btm2.70149

**Published:** 2026-05-19

**Authors:** Dezhen Mou, Minghan Yang, Mengnan Jia, Yuhan Liu, Jun Chen, Xinye Xiao, Ming Chen

**Affiliations:** ^1^ Department of Marine Biological Science & Technology, College of Ocean & Earth Sciences Xiamen University Xiamen China; ^2^ State‐Province Joint Engineering Laboratory of Marine Bioproducts and Technology Xiamen University Xiamen China; ^3^ The College of Materials Xiamen University Xiamen China; ^4^ Fujian Ocean Innovation Center Xiamen China; ^5^ Marinesharp Therapeutics Co. Ltd. Xiamen China; ^6^ State Key Laboratory of Vaccines for Infectious Diseases Xiang An Biomedicine Laboratory Xiamen China

**Keywords:** hollow sponge *Haliclona* sp. spicules, mathematical model of skin penetration, ovalbumin, skin drug delivery

## Abstract

This study introduces hollow sponge *Haliclona* sp. spicules (oSHS) as an innovative microneedle technology for enhancing the skin delivery of biomacromolecules. oSHS were developed by etching SHS in 2 mol/L NaOH for 48 h, optimizing hollow channel formation while preserving structural integrity. In vitro experiments demonstrated that oSHS significantly enhanced drug absorption efficiency compared to SHS, microneedles, roller microneedles, and iontophoresis alone. Combining oSHS with iontophoresis yielded a synergistic effect, increasing biomacromolecule delivery by up to 35.09% (*p* <0.05). In vivo skin delivery of ovalbumin (OVA) via oSHS elicited immune responses comparable to subcutaneous injection, with elevated OVA‐specific IgE and IL‐4 levels. A mathematical model was developed to describe oSHS‐mediated permeation, revealing dose‐dependent delivery and uniform distribution of biomacromolecules in deep skin layers, contrasting with localized accumulation from injections. Safety assessments confirmed minimal and reversible skin irritation, resolving within 72 h. These findings highlight oSHS as a safe, efficient, and scalable strategy for skin delivery of proteins, nucleic acids, and other macromolecules, offering significant potential for non‐invasive therapeutic applications.

**Translational Impact Statement**

This study presents hollow sponge spicules (oSHS) as a novel, non‐invasive microneedle system that significantly enhances the skin delivery of proteins and antibodies. By enabling uniform and dose‐dependent penetration through the skin, oSHS effectively delivered OVA and elicited immune responses comparable to injections. This safe, scalable strategy holds strong translational potential for painless, needle‐free administration of macromolecular therapeutics, offering a patient‐friendly alternative for clinical vaccination and biologic drug delivery.

## 1 INTRODUCTION

Biomacromolecules, such as proteins, peptides, and nucleic acids, offer unique advantages in disease treatment and health promotion due to their specialized biological functions and pharmacological activities.^1^, ^2^, ^3^ Traditional drug administration methods, mainly including oral, injection, and inhalation routes, have their limitations, such as first‐pass metabolism and the lack of patient acceptance and compliance.^4^ Skin drug delivery aims to directly deliver therapeutics into the human body through the skin barrier, providing a non‐invasive route of administration,^5^, ^6^ which is of great significance in improving patient compliance,^7^ reducing systemic side effects, and among other.^8^, ^9^ However, large molecular sizes of biomacromolecules and their limited permeability often hinder skin absorption and effectiveness. Consequently, achieving direct delivery of these biomacromolecules through the skin for enhanced therapeutic impact has become a prominent area of research.^10^

Current strategies to enhance drug penetration include the use of penetration enhancers, nanocarriers, microneedle patches, and other advanced technologies.^11^, ^12^, ^13^, ^14^ These approaches modify drug properties and skin permeability, improving penetration and absorption rates.^15^, ^16^, ^17^ Currently, microneedles (MNs) are classified into five main types: solid, hollow, coated, dissolving, and hydrogel MNs.^18^ These systems offer several advantages, including minimally invasive administration, high drug delivery efficiency, broad adaptability, and effective controlled‐release properties.^19^ However, due to the self‐healing nature of the skin, the microchannels created by MNs close rapidly after insertion. Although dissolving and hydrogel MNs can extend the retention and release time of drugs in the skin, their application is still largely limited to small or specific areas due to the patch size and the requirement for skin smoothness. In our previous studies, we identified that sponge *Haliclona* sp. spicules (SHS) exhibit a permeability‐enhancing effect similar to MNs.^20^, ^21^, ^22^ SHS significantly increases skin permeability, facilitating the entry of drug molecules, and offers unique advantages over conventional skin delivery methods. These include the rapid formation of numerous nanometer‐sized microchannels, applicability to non‐flat skin areas, and natural dislodgement within 72 h. However, the nanometer‐sized channels created by SHS are insufficient for the efficient delivery of biomacromolecules. To address this limitation, we propose improving SHS by constructing micrometer‐sized hollow channels, thereby enhancing the delivery efficiency of biomacromolecules.

In this study, we fabricated the hollow SHS (oSHS) and evaluated its effectiveness for skin delivery of biomacromolecules compared to SHS, MNs (MN), roller MNs, and iontophoresis. We also combined oSHS with iontophoresis to further improve the skin absorption of biomacromolecules. We next successfully used oSHS to deliver the ovalbumin (OVA) in vivo. Additionally, we conducted a preliminary analysis of the relationship between the drug's physicochemical properties and delivery efficiency under oSHS treatment. The overall concept of oSHS for biomacromolecule delivery is illustrated in Figure 1.

**FIGURE 1:**
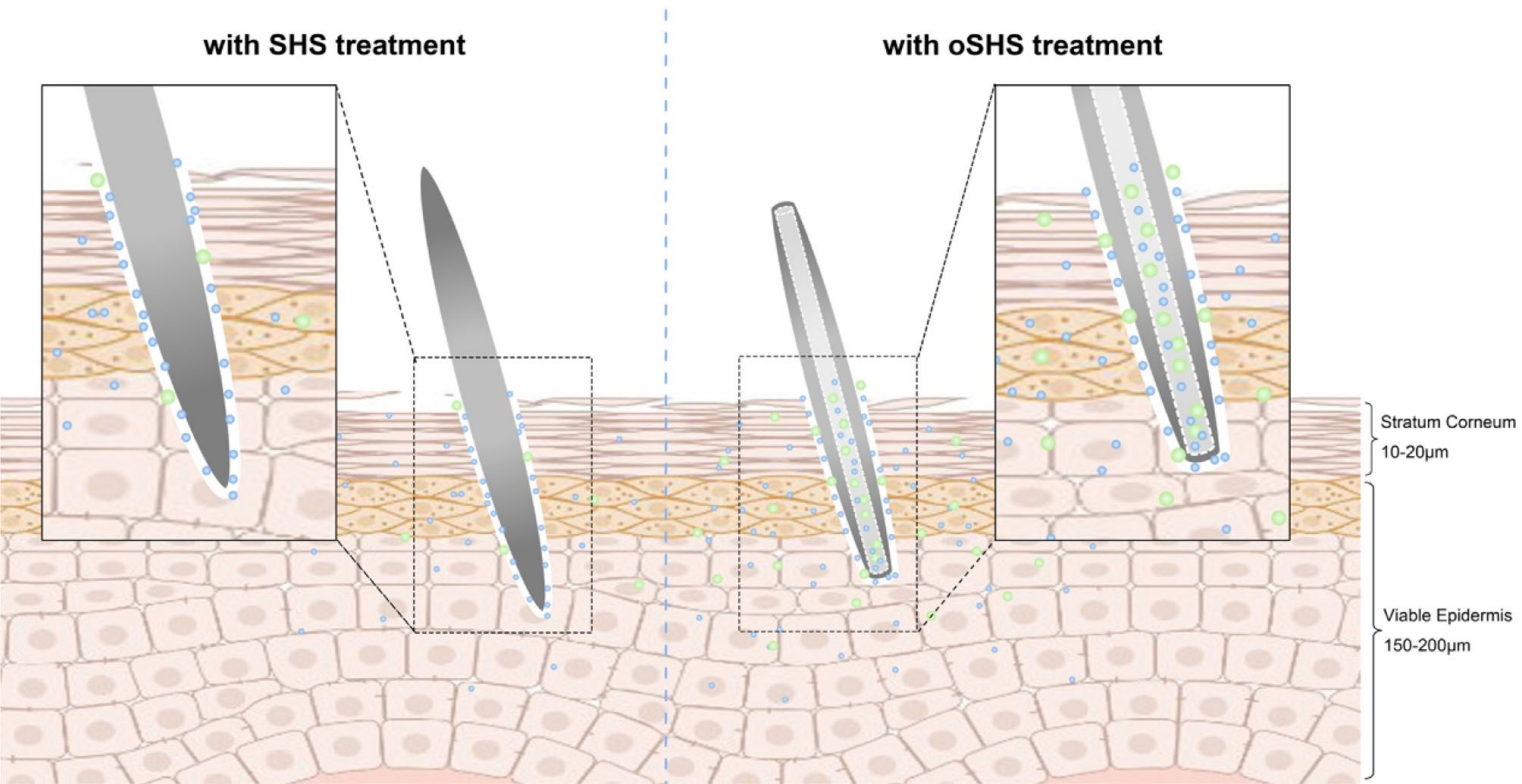
Schematic illustration of hollow sponge spicules for topical application.

## 2 MATERIALS AND METHODS

### 2.1 Chemicals and animals

Fluorescein sodium (FS), Chitosan, poly vinyl alcohol (PVA), and Polyvinyl pyrrolidone (PVP) were sourced from Sigma‐Aldrich (St. Louis, Missouri, USA). Fluorescein isothiocyanate‐dextran (FD) with different molecular weights (FD 20K, FD 40K, and FD 150K) were purchased from Acmec Biochemical Co., Ltd. (Shanghai, China). Dextran (DEX) was purchased from aladdin (Shanghai, China). Trypan was purchased from solarbio (Beijing, China). OVA, mouse IL‐4 ELISA kit, and mouse OVA sIgE (Ovalbumin Specific IgE) ELISA kit were sourced from Ruida Henghui Technology Development Co., Ltd. (Beijing, China). SHS was obtained from Marinesharp Therapeutics Co., Ltd. (Xiamen, China). All other chemicals were of analytical grade. This study utilized female guinea pigs (6–8 weeks old), housed under strict sterile conditions at the Experimental Animal Center of Xiamen University (Xiamen, China). All animal experiments were performed under the ethical approval (No. XMULAC20240004) issued by Xiamen University's Institutional Animal Care Committee, in strict accordance with China's Laboratory Animal Administration Regulations.

### 2.2 Preparation and characterization of oSHS

Purified SHS were immersed in a sodium hydroxide (NaOH) solution with different concentrations (0.5, 1, and 2 mol/L) by using an electric shaker for a specified duration (0.75, 1.5, 24, 48, and 72 h) to selectively dissolve their tips, resulting in the formation of oSHS. The obtained oSHS were neutralized to a pH of 7 using L‐ascorbic acid, followed by rinsing with ultrapure water (MilliQ) using a chromatography column (AC10885, Acmec, China) for 72 h.

oSHS were characterized using optical microscopy (AXIO Image A2®, Zeiss, Germany) and field emission scanning electron microscopy (SEM) (Quanta 650 FEG, FE company, USA) to determine their opening size and opening rate. To assess the continuity of the internal channel, SEM cross‐sectional images were acquired at the tip, mid‐shaft, and base of the spicules. Nile Red staining was performed on oSHS and observed under confocal microscopy (LSM780, Zeiss, Germany) by introducing a Nile Red ethanol solution to the oSHS and analyzing its fluorescence distribution. Low‐frequency ultrasonic impact (XO‐650D, ATPIO, China) was applied to an aqueous solution containing oSHS (0.1 g/mL) to evaluate their mechanical strength by observing their structural integrity.

### 2.3 Preparation of MN patches

The MN patch was fabricated using two‐step molding technology from 0.9 × 0.9 cm polydimethylsiloxane (PDMS) molds that comprised a 10 × 10 array with an 820 μm height and a 280 μm base diameter. The tip of MNs was prepared from a mixed solution containing 2% w/w chitosan, 17% w/w PVA, and 35% w/w DEX. Firstly, to prepare the MN tip, the PDMS mold was filled with the mixed solution, centrifuged at 4000 rpm for 1 h, and then dried for 20 min. After that, the PDMS mold was filled with the PVP solution (33.6% w/w) to prepare the MN substrate. The MN substrate was dried overnight and demolded to obtain a MN patch. For MN@PDL1‐vNAR patches, PDL1 vNAR was added to the MN tips solution, according to the preparation of the MN patch.

### 2.4 Characterization of MN patches

#### 2.4.1 Morphological investigation

A digital camera and Motic PA53 FS6 Intermediate fluorescence illumination system (Xiamen, China) were used to assess the visual appearance and the shape of MNs tip, respectively.

#### 2.4.2 Mechanical properties test

The insertion capacity of MN patch was tested on a mouse skin. Specifically, the MN patch was inserted into the mouse skin by pressing against its backing layer with the thumb for 1 min. To verify the successful penetration, the skin was stained with 0.4% trypan blue for 10 min after removing the patch and thoroughly washed with PBS. Images of the penetrated skin site with trypan blue staining were captured using a digital camera (Nikon D3100).

Afterward, the mechanical properties of the MN patches were measured using a universal testing machine (Microtester 5948, Instron, USA). The MN patches were fixed on the universal testing machine with the tips facing upward, and the breaking strength of the needle tip was measured at a test speed of 0.5 mm/min.

### 2.5 Skin penetration of biomacromolecules in vitro

Fresh porcine skin (purchased from Yinxiang Group, Xiamen, China) was prepared for skin penetration experiment in vitro. Hair was trimmed to a length of no more than 2 mm using an electric shaver (Yizu DDG‐S02, Zhejiang, China). Porcine skin samples underwent cryopreservation at −80°C, followed by thawing and sectioning into 36 mm diameter discs using a circular punch. Hypodermal adipose tissue was surgically excised using sterile blades, followed by extensive irrigation of the dermal surface with 0.9% NaCl solution. The prepared skin samples were mounted into vertical Franz diffusion cells (FDCs, Zhengtong, Tianjin, China).

Afterwards, different groups were subjected to different treatments in accordance with the requirements of the experiment. For the SHS or oSHS group, a predetermined dose of SHS or oSHS was applied to the skin, followed by a 2‐min electrical massaging with an electric massager operating at 0.3 N force and 300 rpm frequency, and then the drug solution was applied to the skin surface. In the SHS or oSHS + ITP group, the drug solution was topically administered following dermal massage, the applied current density was 0.5 mA/cm^2^, and the time was 15 min. For the MN group, MN@PDL1‐vNAR patches were pressed for 5 min. For the roller MN group, a dermaroller device (model HC902, 0.2 mm, 162 MNs, Munich, Germany) was used to treat the dermal surface, rolled horizontally, vertically, and diagonally across the skin for 8 times, then the drug solution was administered to the surface of the skin. For the injection group, the drug solution was injected into the porcine skin using a syringe. The test drug solution was FS (500 μL, 2 mg/mL), and the other test solutions included fluorescein isothiocyanate‐dextran with different molecular weights (200 μL, 1 mg/mL) or PDL1 vNAR (200 μL, 1 mg/mL). All skin samples were subjected to incubation on Franz diffusion cells at 37°C for a period of 16 h, with moderate stirring. Skin samples and the solution from the receiving chamber were collected to measure the concentration of these testing biomacromolecules.

To determine the distribution of the testing biomacromolecules within the skin, the stratum corneum (SC), viable epidermis, and dermis were separated using tape stripping and extraction methods described in our previous work.^21^ These layers were soaked in a methanol/PBS solution for 24 h, and the resulting extracts were analyzed for the testing biomacromolecules. The content of sodium fluorescein, FD 20 K, FD 40 K, FD 150 K and CY5.5‐PDL1 vNAR in the collected solution was detected by a fluorescence spectrometer (Tecan Infinite 200PRO, Tecan Trading AG, Switzerland). Sodium fluorescein exhibits absorption and fluorescence emission maxima at 485 nm and 535 nm, respectively. In 0.2M PBS, the standard curve is $y=448.64\;x+300.32,\;R^{2}=0.9997$. In methanol/PBS, the standard curve is $y=420.96x+77.159,\;R^{2}=0.9993$. Fluorescent glucan demonstrated an absorption peak at 490 nm and emitted fluorescence at 530 nm. In methanol/PBS and citric acid/sodium citrate solutions, the standard curves of FD 20 K are $y=789.38x-79.589,\;R^{2}=0.9991；y=10.933x+3.8464,\;R^{2}=0.9996.$ And FD 40 K's are $y=7.8474x-58.337,\;R^{2}=0.9993;y=0.4189x+10.959,\;R^{2}=0.9994$. And FD 150 K's are $y=807.14x+12.404,\;R^{2}=0.9996;y=9.2927x-48.613,\;R^{2}=0.9994$. The levels of OVA‐sIgE and IL‐4 in the collected blood were determined by a detection kit, the standard curves are $y=11.526x-2.3014,\;R^{2}=0.9946$ and $y=72.851x-26.31,\;R^{2}=0.9912.$ Cy5.5 exhibits absorption and fluorescence emission maxima at 675 and 694 nm, respectively. In methanol/PBS and citric acid/sodium citrate solutions, the standard curve of CY5.5‐PDL1 vNAR are $y=8511.3x+438.13,\;R^{2}=0.999$; $y=11969x+245.83,\;R^{2}=0.9997$.

### 2.6 Skin delivery of OVA in vivo

Guinea pigs (8–12 weeks old, female; *n* = 3 per group) were anesthetized with isoflurane using a gas anesthesia machine, and the fur around the elbow was carefully shaved. A 1 cm^2^ area was marked on the skin, followed by SHS and oSHS treatments applied separately. OVA solution (100 μL of 35 mg/mL OVA saline solution) was then topically applied, followed by delivery for consecutive days. The status of the immune response of the guinea pigs should be observed and recorded on a daily basis, according to the judgment criteria in the appendix of the Chinese Pharmacopeia 2010. On day 8, venous blood samples were collected to assess the concentrations of OVA‐specific immunoglobulin E (S‐IgE) and IL‐4 by ELISA kit.

### 2.7 Skin irritation test of oSHS

We selected guinea pigs (8–12 weeks old, female; *n* = 3 per group) as the experimental subjects. After anesthetizing them with isoflurane, we shaved the hair on their backs and marked a 1 cm^2^ area on the back skin. Different concentrations of oSHS solution (2 mg/cm^2^, 10 mg/cm^2^) were massaged onto the marked area on the guinea pigs' backs, followed by rinsing with physiological saline, with the procedure repeated three times. Photographs of the skin were taken at 0, 24, 48, and 72 h post‐treatment, and skin irritation was evaluated using the method of Draize.^23^ Additionally, skin tissues were collected at 24, 48, 72, and 240 h post‐treatment, and HE‐stained sections were prepared; immune cells in the sections were counted using IPWIN 32.

### 2.8 Data analysis

The combinatorial enhancement coefficient (SI) was mathematically expressed as follows:

$$\mathrm{SI}=\frac{J_{\text{comb}}}{\sum J_{\text{isol}}}$$

where *J*_comb_ represents the total transdermal flux from enhancer combinations, and *J*_isol_ denotes the permeation achieved by individual enhancers.

Data are represented as mean ± standard deviation (SD). Intergroup differences were evaluated through two‐tailed Student's *t*‐tests, with statistical significance set at *p* <0.05. Orthogonal design datasets were analyzed using IBM SPSS Statistics 23.0 (IBM Corp., Armonk, NY, USA) for multifactorial modeling.

## 3 RESULTS

### 3.1 Preparation and characterization of oSHS

We utilized NaOH solution under varying preparation conditions to dissolve the tips of SHS, thereby forming hollow channels within oSHS. The formation of these channels was optimized by evaluating the opening rate, channel size, and structural integrity of oSHS (Figure 2a,b). The results showed that treatment with 2 mol/L NaOH for 72 h yielded a significantly higher opening rate and larger channel size compared to all other groups (*p* <0.05). However, this condition also caused a marked reduction in the structural integrity of oSHS (Figure 2c). Therefore, the optimal preparation condition for oSHS was determined to be immersion in 2 mol/L NaOH for 48 h. SEM analysis further demonstrated that the hollow structure was not restricted to the tip region. Cross‐sectional images obtained at the tip, mid‐shaft, and base consistently revealed a continuous hollow channel (Figure 2d). This morphology may reflect preferential etching along the axial core region, which is known to contain a proteinaceous filament in siliceous sponge spicules.^24^ Additionally, Nile Red staining analyzed via confocal microscopy also demonstrated the stable construction of internal hollow channels, which were shown to facilitate the entry of the testing compound (Figure 2e).

**FIGURE 2:**
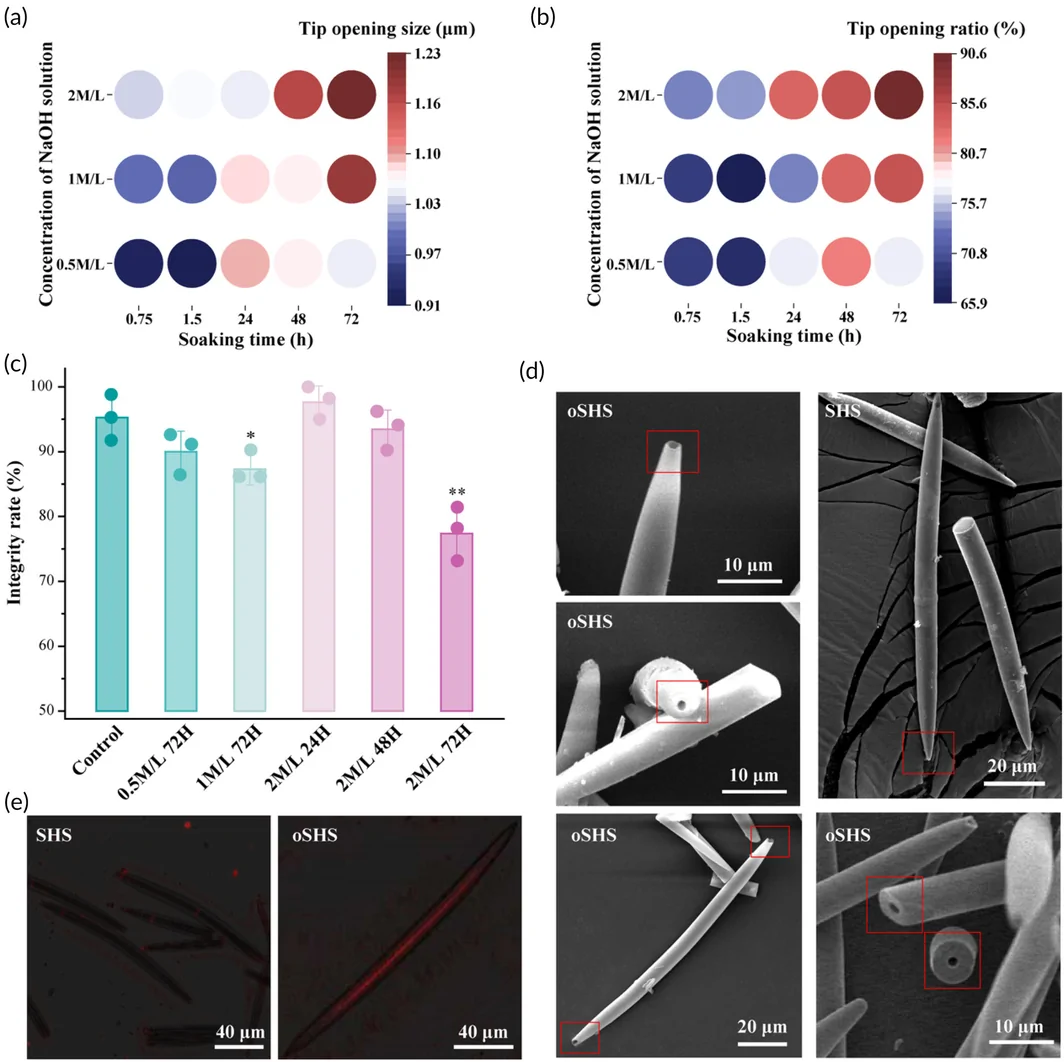
(a) Comparison of the results of different concentrations of NaOH on the opening size of SHS treated with different durations and untreated SHS; (b) Comparison of the results of different concentrations of NaOH on the opening rate of SHS treated with different durations and untreated SHS; (c) Integrity of SHS treated with NaOH solutions after ultrasonic. The data values are mean ± SD (*n* = 3), *: *p* <0.05, **: *p* <0.01; (d) Visualization and characterization of oSHS and SHS; (e) Confocal image of oSHS and SHS.

Up to this point, the optimal preparation conditions that maximized the formation of hollow channels while preserving the mechanical integrity necessary for effective skin penetration after alkaline treatment have been determined. This novel biomaterial represents a promising alternative to artificial MNs, with significant potential for future applications and market opportunities.

### 3.2 Skin penetration of biomacromolecules using oSHS in vitro

The transdermal permeation and cutaneous retention of multiple model compounds were systematically compared across diverse enhancement modalities: SHS, oSHS, and their combinatorial use with iontophoresis (ITP) in ex vivo models. For sodium fluorescein (logP = −1.8, MW = 376.36), oSHS alone and oSHS combined with ITP (oSHS+ITP) achieved total skin absorption rates of 24.08% ± 1.84% and 42.67% ± 4.12%, respectively, both significantly higher (*p* <0.05) than those of all other groups (Figure 3a). Moreover, a synergistic effect was observed between SHS and ITP (synergistic index [SI]: 1.35 ± 0.04) and between oSHS and ITP (SI: 1.44 ± 0.21) on SF skin absorption (Figure 3b). The skin penetration behavior of SF under different penetration‐enhancing strategies was further visualized using confocal microscopy (Figure 3c).

**FIGURE 3:**
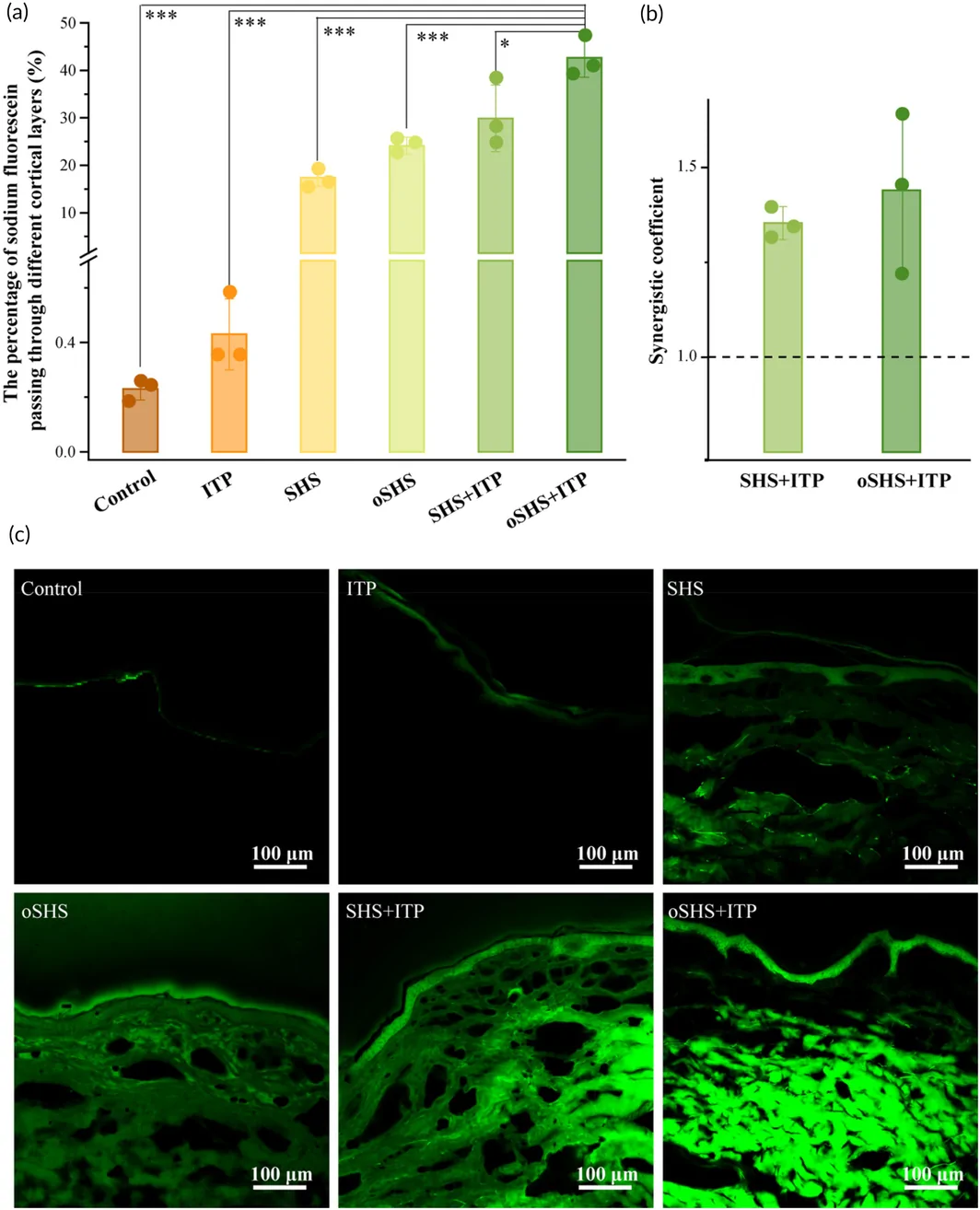
(a) Absorption rate of fluorescein sodium in different skin layers after in vitro percutaneous delivery experiment. The data value is the mean ± SD (*n* = 3), where *: *p* <0.05, ***: *p* <0.001. (b) Synergistic coefficient of percutaneous delivery of FNA using different permeation promoting methods. (c) In vitro percutaneous delivery fluorescence distribution map of sodium fluorescein.

In the case of FD 20 K, oSHS alone and oSHS combined with ITP (oSHS+ITP) achieved total skin absorption rates of 7.69% ± 1.72% and 35.09% ± 12.00%, respectively, both significantly higher (*p* <0.05) than those of all other groups (Figure 4a). While a synergistic effect was observed between oSHS and ITP (SI: 3.29 ± 0.56) on FD 20 K skin absorption, there was no synergistic effect between SHS and ITP (SI: 0.81 ± 0.02) (Figure 4b). The skin penetration behavior of FD 20 K under different penetration‐enhancing strategies was further visualized using confocal microscopy (Figure 4c). We also observed similar results in the case of FD 40 K and FD 150 K (Supplementary Figures 1, 2 for FD 40 K and Supplementary Figures 3, 4 for FD 150 K). The in vitro studies showed a marked increase in drug absorption efficiency, particularly in the dermis and epidermis layers, when treated with oSHS. And the combination of oSHS with iontophoresis produced a strong synergistic effect, significantly enhancing dermal absorption efficiency, particularly in the dermis and epidermis layers.

**FIGURE 4:**
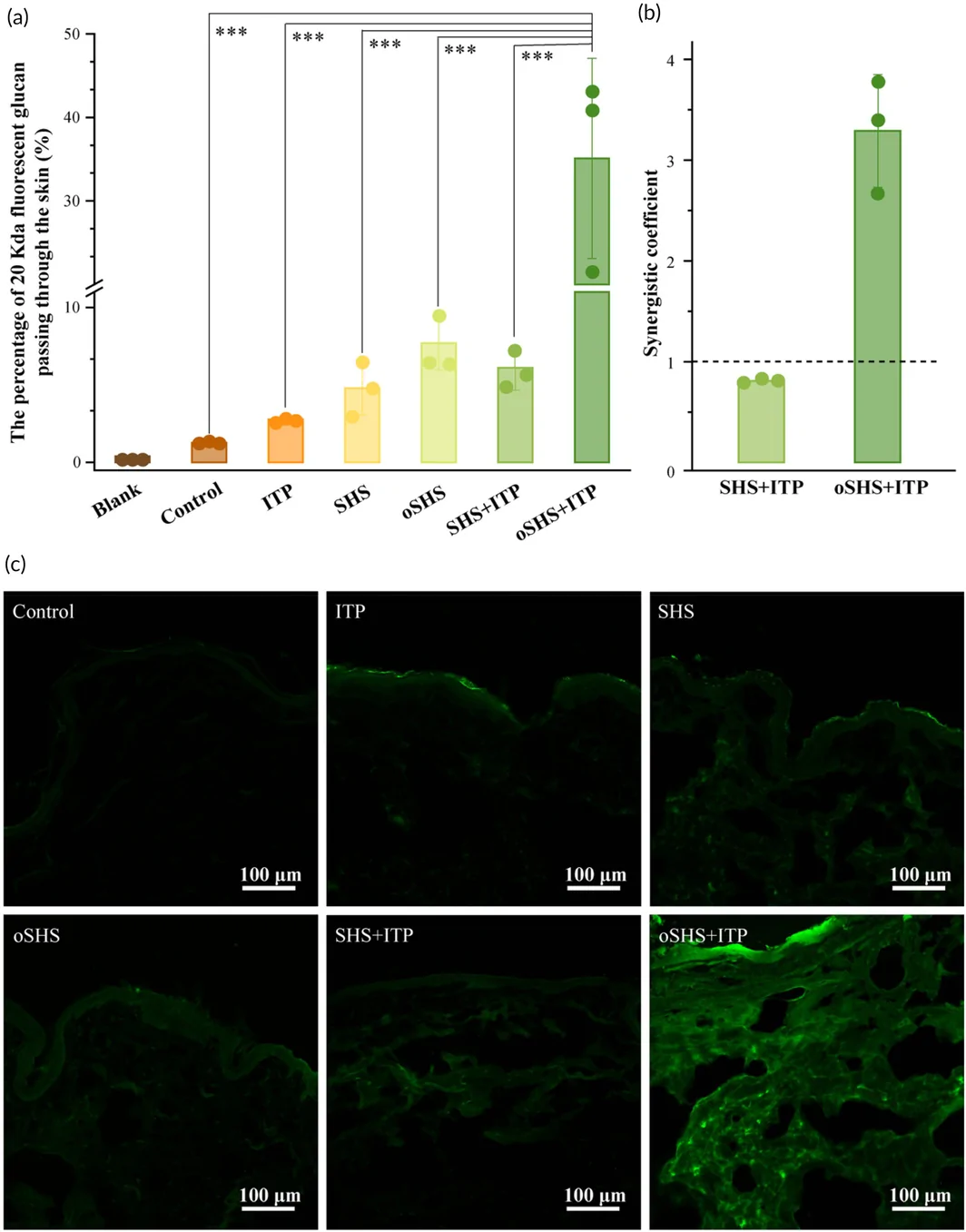
(a) Comparison of in vitro percutaneous extraction of model molecules (FD 20 K) using ITP, SHS, oSHS, SHS + ITP, and oSHS+ITP. The data value is the mean ± SD (*n* = 3), where ***: *p* <0.001. (b) Synergistic coefficient of percutaneous delivery of FD 20 K using different permeation promoting methods. (c) In vitro percutaneous delivery fluorescence distribution of FD 20 K.

We observed that the penetration of FD 150 K into the skin was directly proportional to the dosage of oSHS applied, indicating a dose‐dependent enhancement of biomacromolecule delivery by oSHS (Figure 5). Furthermore, biomacromolecules delivered topically using oSHS were evenly distributed within the skin. To further verify the distribution of FD 150 K after oSHS treatment, dermal samples were collected from three random sites within the treated area. No significant differences in FD 150 K concentration were observed among these sites, indicating a relatively uniform distribution throughout the treated region (Figure S6). In contrast, subcutaneous injection resulted in biomacromolecule accumulation at the injection center, with no detectable distribution at distances of 5 mm or 1 cm from the injection site (Figure 5). The enhanced efficiency reduced the time required for iontophoresis treatments, improved the safety profile of the delivery system, and offered a more effective approach for delivering hydrophilic charged drugs dermally.

**FIGURE 5:**
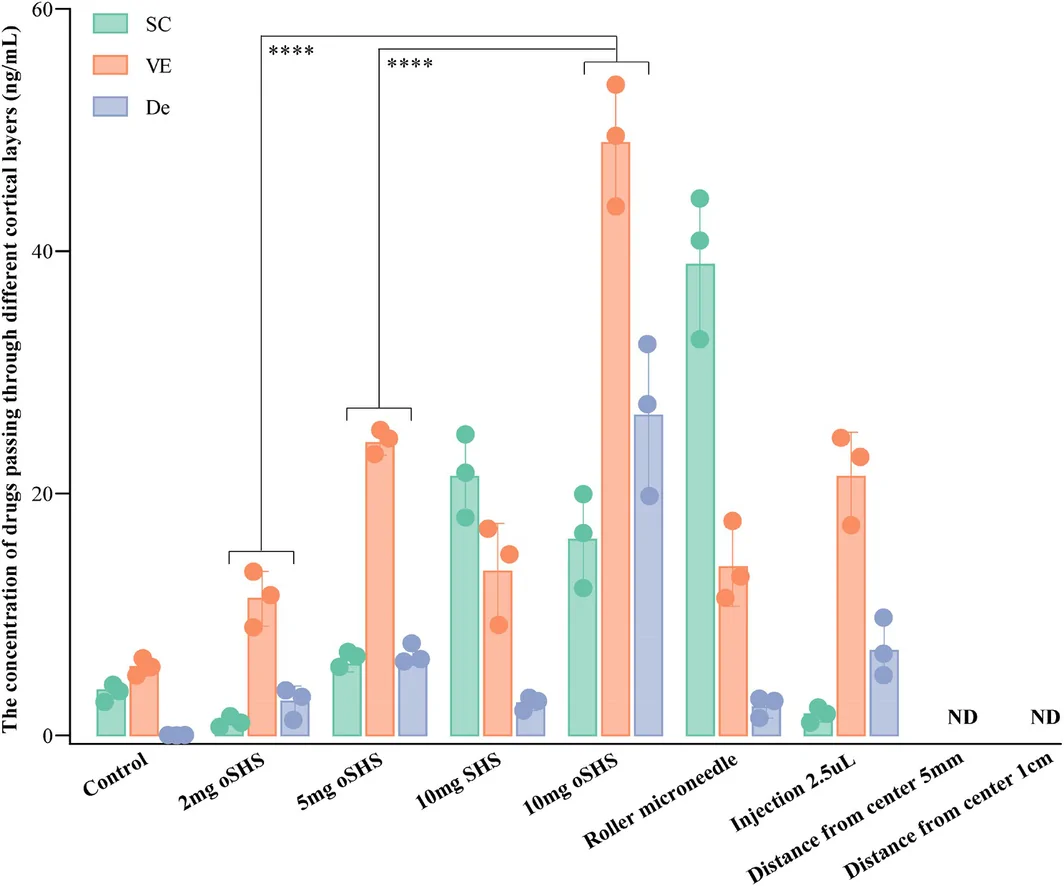
Comparison of delivery efficiency of 10 mg SHS, 2 mg oSHS, 5 mg oSHS, 10 mg oSHS, roller MNs, and injection of in vitro percutaneous delivery model molecules (150 kDa fluorescent glucan) in different cortical layers. ND: Not detectable. The data values are the mean ± SD (*n* = 3), where ****: *p* <0.0001, with a distance of 5 mm and 1 cm from the center for the injection group. Samples were taken separately from different locations of the injection center, and the dosage for the injection group was 2.5 μL.

Antibody‐based therapeutics have been authorized for treating a wide range of serious human diseases. However, the poor permeability of these therapeutics through the SC limits their application. In addition to the previously tested fluorescein isothiocyanate‐dextran series, we further evaluated the effectiveness of oSHS for skin delivery of shark anti‐PDL1 variable new antigen receptor (PDL1 vNAR) compared to MNs. Here, we fabricated MN patches using a two‐step process. The topography of the MN patches obtained by demolding was a neatly arranged array of 10 × 10 MNs (Figure 6a). The needle tips of the MN patches are quadrangular pyramid, complete, uniform, and sharp with a height of approximately 820 μm, a base diameter of 280 and 500 μm in interneedle spacing (Figure 6b,c). The MN was inserted into the mouse skin and subsequently stained with trypan blue, the locality of trypan blue staining was consistent with the insertion sites of MN array, demonstrating successful skin piercing (Figure 6d). The mechanical properties of MN patches are evaluated through the compression test (Figure 6e). An individual MN can tolerate a maximum compressive force of∼0.8 N on average, which was significantly greater than the minimum force required to pierce skin (0.1 N).^25^ Finally, we investigated the effect of oSHS and MN patches on the PDL1 vNAR skin absorption. The utilization of oSHS (25.19% ± 3.03%) significantly enhanced the total skin absorption of PDL1 vNAR compared to both the control (5.84% ± 1.88%) and the MN group (11.47% ± 2.72%) (Figure 6f). These findings have demonstrated that the administration of PDL1 vNAR based oSHS significantly increase the absorption of PDL1 vNAR into deep skin compared to control. Together, these results highlight the efficacy of oSHS in overcoming the SC barrier for the delivery of antibody drugs.

**FIGURE 6:**
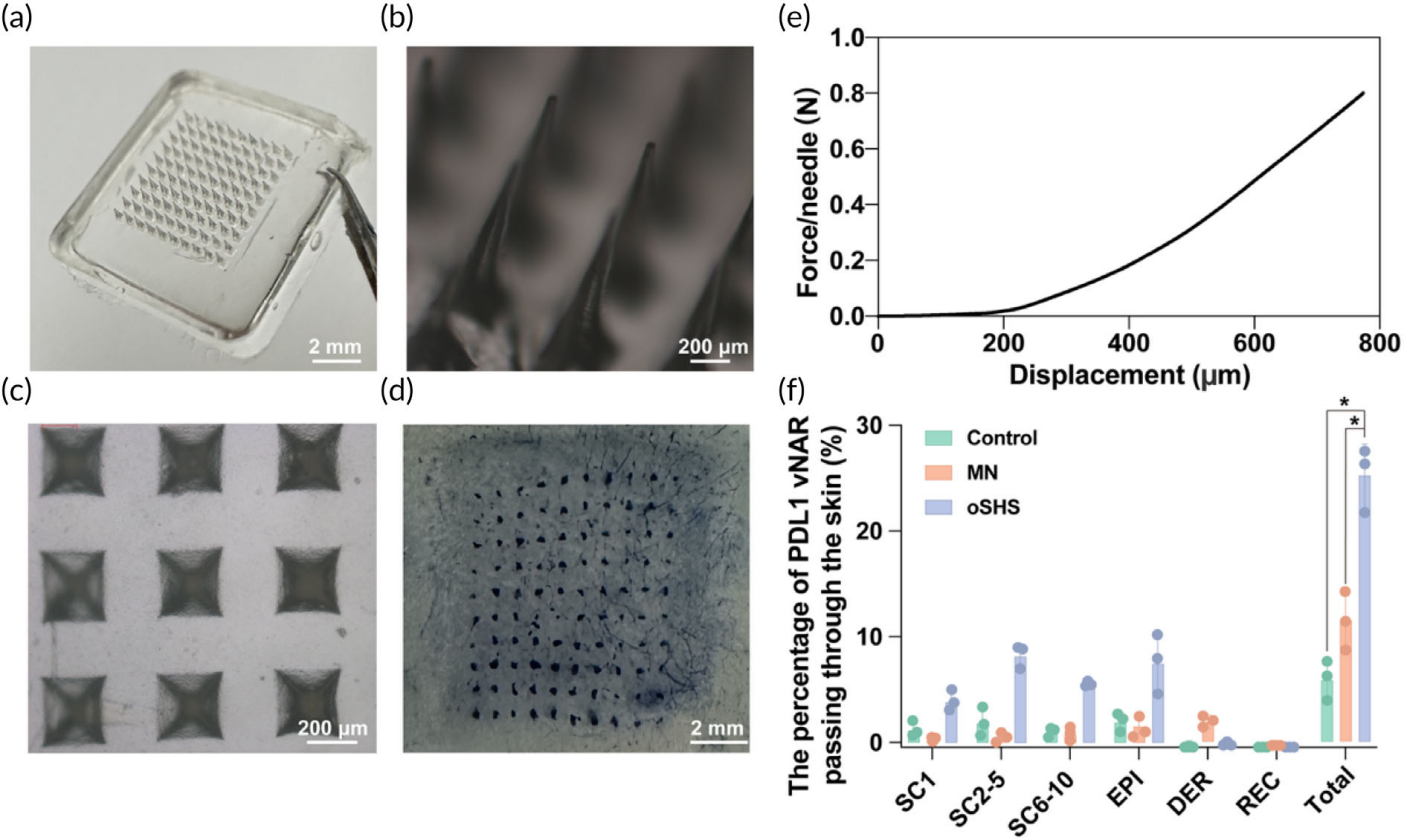
(a) Camera image of the MN; (b) Micro‐graph of the MN (Scale bar: 200 μm). (c) Top view of the MN (scale bar = 200 μm). (d) A photograph of mouse skin stained with trypan blue after microneedle insertion. (e) Mechanical analysis of the MN. (f) Comparison of delivery efficiency of oSHS and MN in vitro percutaneous delivery CY5.5‐PDL1 vNAR in different cortical layers. The data values are the mean ± SD (*n* = 3), where *: *p* <0.05.

### 3.3 Mathematical model of skin penetration behavior using oSHS

Previous studies have demonstrated that skin penetration models for intact SC follow Fick's first law^26^:

$$\log k_{p}=a+b\log P-c\mathrm{MW}$$

where $k_{p}$ is the skin permeability, a, b, c are constants, P is oil and water distribution coefficient of the target compounds, MW is molecular weight of the solute.

Alexey S. Rzhevskiy et al.^27^ extended this concept to microporous skin, proposing the following model for drug permeation:

$$k_{p}\approx 0.36n_{p}r_{p}\log{\mathrm{MW}}^{-0.6}$$

where $k_{p}$ is the skin permeability, $r_{p}$ is the pore radius, $n_{p}$ is the pore density, MW is molecular weight of the solute.

In this study, we developed a mathematical model for skin treated with oSHS. Unlike MNs, which are removed after application and leave “funnel‐shaped” perforation channels, oSHS remains embedded in the skin during treatment. This creates annular channels and hollow channels within the skin. The central hollow region formed by oSHS has a radius $r_{p}$, while the surrounding solid structure retains the original radius of the SHS $r_{s}$, as illustrated in Figure 7.

**FIGURE 7:**
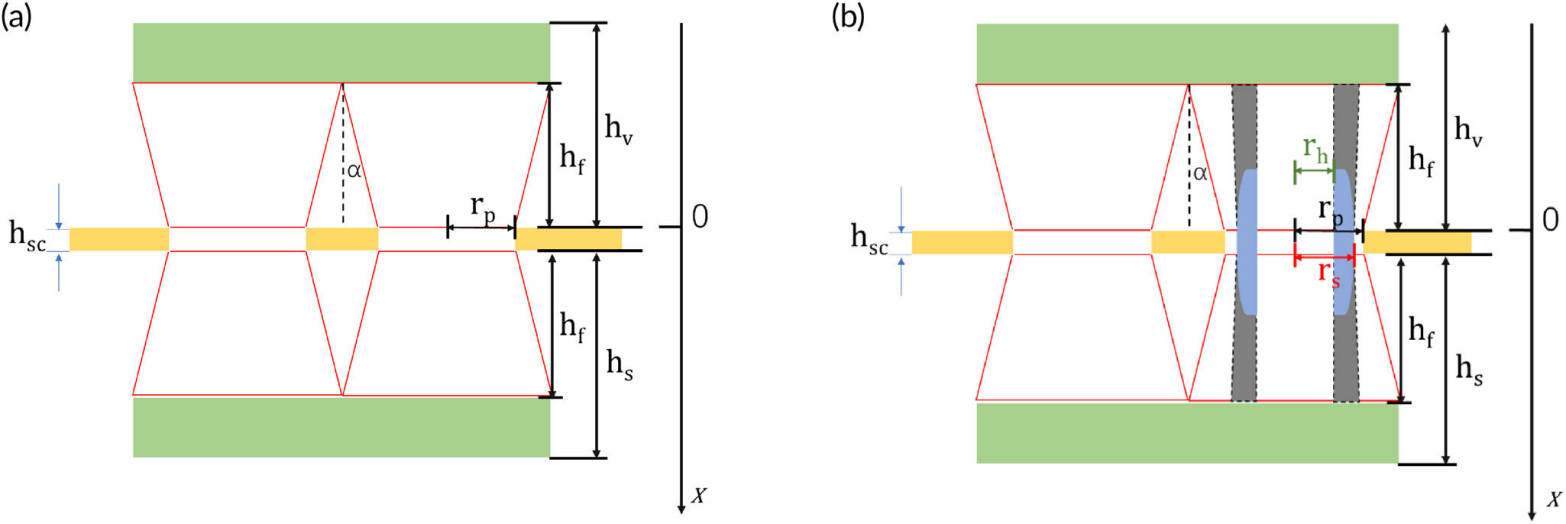
(a) “Perforated” skin model formed by microneedles; (b) Annular channel skin model formed by oSHS.

Similarly, oSHS form annular channel skin models in diffusion resistance

$$R=\int_{-h_{v}}^{h_{\mathrm{sc}}+h_{s}}\frac{\mathrm{dx}}{K\left(x\right)D\left(x\right)a\left(x\right)}$$

Which $K\left(x\right)$ and $D\left(x\right)$ describe the distribution and diffusion of solute in different phase, in solvent $K\left(x\right)=1$, $D\left(x\right)=D_{v}$, in the skin $K\left(x\right)=K_{s}$, $D\left(x\right)=D_{s}$. Assumptions on the cuticle of the hole is filled with solvent, the distribution and the diffusion coefficient of the solute in the hole are the same in the solvent, $K\left(x\right)=1$, $D\left(x\right)=D_{v}$.

$a\left(x\right)$ is the function of diffusion area in 𝑥 direction. When $0\leq x\leq h_{\mathrm{SC}}$, $a\left(x\right)=n_{p}\pi \left(r_{p}^{2}-r_{s}^{2}\right)$. In the “funnel,” $\;a\left(x\right)=\left(1-\frac{r_{s}^{2}}{r_{p}^{2}}\right)\cdot n_{p}\pi r_{f}^{2}\left(x\right)$. The $r_{f}$ of both skin sides, respectively, represents as $\;r_{f}\left(x\right)=r_{p}+4\left(x-h_{\mathrm{SC}}\right)/\pi$ and $r_{f}\left(x\right)=r_{p}-4x/\pi$.

The $\left(x\right)$, $D\left(x\right),\;\text{and}\;a\left(x\right)$ function can be expressed as:

$$K\left(x\right)=\left\{\begin{array}{c} 1,\;-h_{v}\leq x\leq h_{\mathrm{SC}}\\ K_{s},\;x>h_{\mathrm{SC}} \end{array}\right.$$

$$D\left(x\right)=\left\{\begin{array}{c} D_{v},\;-h_{v}\leq x\leq h_{\mathrm{SC}}\\ D_{s},\;x>h_{\mathrm{SC}} \end{array}\right.$$

$$a\left(x\right)=\left\{\begin{array}{c} 1,\;-h_{v}\leq x\leq -h_{f}\\ \left(1+\frac{r_{h}^{2}}{r_{p}^{2}}-\frac{r_{s}^{2}}{r_{p}^{2}}\right)\cdot n_{p}\pi {\left(r_{p}-4x/\pi \right)}^{2},\;-h_{f}<x\leq 0\;\\ n_{p}\pi \left(r_{p}^{2}-r_{s}^{2}\right),\;0<x\leq h_{\mathrm{SC}}\\ \left(1+\frac{r_{h}^{2}}{r_{p}^{2}}-\frac{r_{s}^{2}}{r_{p}^{2}}\right)\cdot n_{p}\pi {\left(r_{p}+4\left(x-h_{\mathrm{SC}}\right)/\pi \right)}^{2},\;h_{\mathrm{SC}}<x\leq h_{f}+h_{\mathrm{SC}}\\ 1,\;x>h_{f}+h_{\mathrm{SC}} \end{array}\right.$$

By integrating the piecewise function, we can obtain:

$$R=\int_{-h_{v}}^{-h_{f}}\frac{\mathrm{dx}}{D_{v}}+\int_{-h_{f}}^{0}\frac{\mathrm{dx}}{D_{v}\left(1+\frac{r_{h}^{2}}{r_{p}^{2}}-\frac{r_{s}^{2}}{r_{p}^{2}}\right)\cdot n_{p}\pi {\left(r_{p}-\frac{4x}{\pi }\right)}^{2}}+\int_{0}^{h_{\mathrm{sc}}}\frac{\mathrm{dx}}{D_{v}n_{p}\pi \left(r_{p}^{2}-{\;r}_{s}^{2}\right)}$$

$$+\int_{h_{\mathrm{sc}}}^{h_{\mathrm{sc}}+h_{f}}\frac{\mathrm{dx}}{K_{s}D_{s}\left(1+\frac{r_{h}^{2}}{r_{p}^{2}}-\frac{r_{s}^{2}}{r_{p}^{2}}\right)\cdot n_{p}\pi {\left(r_{p}+\frac{4\left(x-h_{\mathrm{sc}}\right)}{\pi }\right)}^{2}}+\int_{h_{\mathrm{sc}}+h_{f}}^{h_{\mathrm{sc}}+h_{s}}\frac{\mathrm{dx}}{K_{s}D_{s}}$$

$$R=\frac{h_{v}-h_{f}}{D_{v}}+\frac{h_{s}-h_{f}}{K_{s}D_{s}}+\frac{h_{\mathrm{sc}}}{D_{v}\left(1+\frac{r_{h}^{2}}{r_{p}^{2}}-\frac{r_{s}^{2}}{r_{p}^{2}}\right)\cdot n_{p}\pi r_{p}^{2}}-\frac{l_{p}-2r_{p}}{4\left(1+\frac{r_{h}^{2}}{r_{p}^{2}}-\frac{r_{s}^{2}}{r_{p}^{2}}\right)\cdot n_{p}l_{p}r_{p}}\left(\frac{1}{D_{v}}+\frac{1}{K_{s}D_{s}}\right)$$

Since the channels formed by oSHS are on the nanoscale, while the hollow ducts at the center of oSHS are micron‐sized, the pore radius is expected to be smaller than the spacing between adjacent pores.

$$R=\left[\frac{1}{D_{v}}\left(1+\frac{4h_{\mathrm{sc}}}{\pi r_{p}}\right)+\frac{1}{K_{s}D_{s}}\right]\frac{1}{4\left(1+\frac{r_{h}^{2}}{r_{p}^{2}}-\frac{r_{s}^{2}}{r_{p}^{2}}\right)\cdot n_{p}r_{p}}$$

$$k_{p}=\frac{1}{R}=4D_{\mathrm{aq}}\left(1+\frac{r_{h}^{2}}{r_{p}^{2}}-\frac{r_{s}^{2}}{r_{p}^{2}}\right)\cdot n_{p}r_{p}{\left[\left(1+\frac{4h_{\mathrm{sc}}}{{\mathrm{\pi r}}_{p}}\right)\frac{D_{\mathrm{aq}}}{D_{v}}+\frac{D_{\mathrm{aq}}}{K_{s}D_{s}}\right]}^{-1}$$

According to the published research,^28^ the permeability coefficient of the annular channel formed by oSHS should approximately be:

$$k_{p}\approx 0.36\left(1+\frac{r_{h}^{2}}{r_{p}^{2}}-\frac{r_{s}^{2}}{r_{p}^{2}}\right)\cdot n_{p}r_{p}{\mathrm{MW}}^{-0.6}$$

The skin treated with oSHS has both annular channels and complete SC, so we think its drug permeability coefficient $k_{p,\text{total}}$ should be the sum of the contribution of the intact SC, $k_{p,\text{complete}\;\mathrm{SC}},$ and the contribution of the annular channel, $k_{p,\;\text{annular channel}}$.

$$\log k_{p,\text{total}}=\frac{1}{m}\log k_{p,\text{complete}\;\mathrm{SC}}+\frac{1}{n}\log k_{p,\text{annular channel}}$$

$$=\frac{1}{m}\left(a+b\log P-c\mathrm{MW}\right)+\frac{1}{n}\log0.36\left(1+\frac{r_{h}^{2}}{r_{p}^{2}}-\frac{r_{s}^{2}}{r_{p}^{2}}\right)\cdot n_{p}r_{p}{\mathrm{MW}}^{-0.6}=a^{\prime }+b^{\prime }\log P+c^{\prime }\mathrm{MW}+d^{\prime }\log{\mathrm{MW}}^{-0.6}$$

Considering that the dose of oSHS used is consistent, we fit the skin permeability, oil–water partition coefficient, and molecular weight of the six target compounds mentioned above, and obtain:

$$\log k_{p}=19.107+0.0000289\mathrm{MW}-1.3501\log P+4.4579\log{\mathrm{MW}}^{-0.6}$$

This analysis allowed us to derive a specialized skin permeation model for oSHS‐mediated drug delivery, offering insights into its application in optimizing dermal therapeutic systems.

### 3.4 Skin delivery of OVA using oSHS in vivo

To further assess the effectiveness of oSHS in delivering macromolecules in vivo, we utilized oSHS for the delivery of OVA and evaluated its potential in eliciting immune responses. Following the different administration of OVA to guinea pigs, a significant immune response was observed on day 2 in both the injection and oSHS groups upon exposure to OVA (Table 1). By days 3 and 8, the immune response in the oSHS group was weaker than that in the injection group but remained significantly stronger than in the control and SHS groups (Table 1).

**TABLE 1:** The occurrence of immune reactions in guinea pigs within 30 min after treatment.

Group	Day 1	Day 2	Day 3	Day 8
Control	×	×	×	×
SHS	×	×	×	Duration of mild dyspnea: 3 min ± 2 min
oSHS	×	Scratching Nose: 2 ± 1 Sneezing: 1 ± 0	Duration of dyspnea: 7 min ± 2 min	Number of head convulsions: 2 ± 1
Injection	×	Scratching Nose: 3 ± 0 Sneezing: 3 ± 1	Duration of dyspnea: 13 min ± 2 min Number of delayed reactions 2 ± 1	Number of convulsions throughout the body: 2 ± 0

Following an 8‐day experimental period, cardiac puncture was performed to obtain blood specimens for analysis of OVA‐specific IgE (sIgE) and IL‐4 concentrations using chemiluminescent immunoassays. IgE is a critical immunoglobulin involved in allergic reactions and immune responses, with sIgE serving as the most direct indicator of immune activation in guinea pigs in this study. IL‐4, a cytokine that regulates T‐cell differentiation and B‐cell‐mediated immune responses, provides a direct reflection of the immune status and inflammation level in the body. The results (Figure 8) demonstrated that oSHS exhibited significant advantages over SHS in delivering OVA through the skin in vivo. Furthermore, IL‐4 levels showed no significant difference between the oSHS and injection groups, highlighting the efficacy of oSHS in antigen delivery.

**FIGURE 8:**
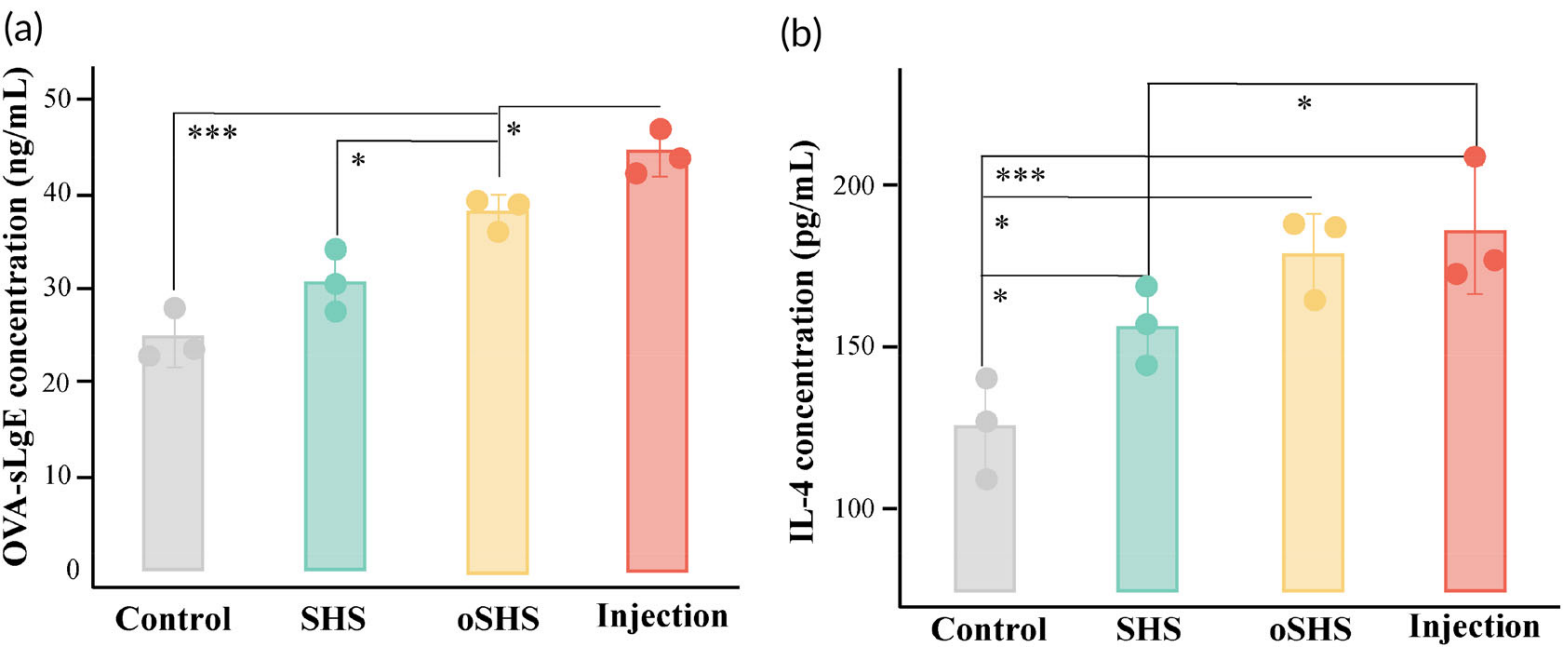
(a) OVA‐sIgE concentration in guinea pigs' serum of different treatment groups. (b) The concentration of IL‐4 in the serum of guinea pigs from different treatment groups. The data value is the mean ± SD (*n* = 3), where *: *p* <0.05, ***: *p* <0.001.

### 3.5 Skin irritation of oSHS

We assessed the potential irritancy of oSHS on the skin of guinea pigs. Following oSHS treatment, mild redness and swelling were observed on the skin, which resolved within 48–72 h (Figure 9a). Subsequent histological analysis revealed a sharp increase in the number of immune cells in the skin post‐treatment, indicating that oSHS activated the skin's immune response. By day 3, the number of immune cells had significantly decreased, and by day 10, it had returned to levels comparable to the control group (Figure 9b). These findings suggest that oSHS induces only mild and reversible acute skin irritation within 10‐day, demonstrating its safety and reliability as a biomaterial for a novel type of MNs. The intensity of skin irritation caused by different treatment methods was subsequently evaluated on guinea pig skin by recording redness, swelling, and edema. The results indicated that the irritancy scores followed the order: control group (0.1) <2 mg/cm^2^ oSHS group (0.7) <10 mg/cm^2^ oSHS group (1.5) (Figure 9c). Both concentrations of oSHS exhibited mild irritation to the skin post‐treatment, demonstrating that oSHS can be safely applied to the skin surface. Following the treatments, skin samples were collected at 0, 24, 48, 72, and 240 h and subsequently stained for analysis (Figure 9d). Immune cell infiltration (leukocytes and/or granulocytes) was observed in the upper dermis after oSHS application. To quantify this response, skin cells within the AOI (area of interest, 450 μm × 150 μm, covering the dermis layer) were analyzed and counted. In the control group, the baseline number of immune cells was 227 ± 30.6. After oSHS application, a robust immune response was observed, with immune cell numbers rising to 551 ± 85.2 at 24 h. At 48 h, the immune cell count remained elevated compared to 24 h, reaching 560.7 ± 62.6. By 72 h, a significant decrease in immune cell numbers was noted, dropping to 409.3 ± 28.6. However, by 240 h, the skin had largely recovered, and the immune cell count decreased to 264.7 ± 17.24, showing no significant difference compared to the control group (*p* >0.05).

**FIGURE 9:**
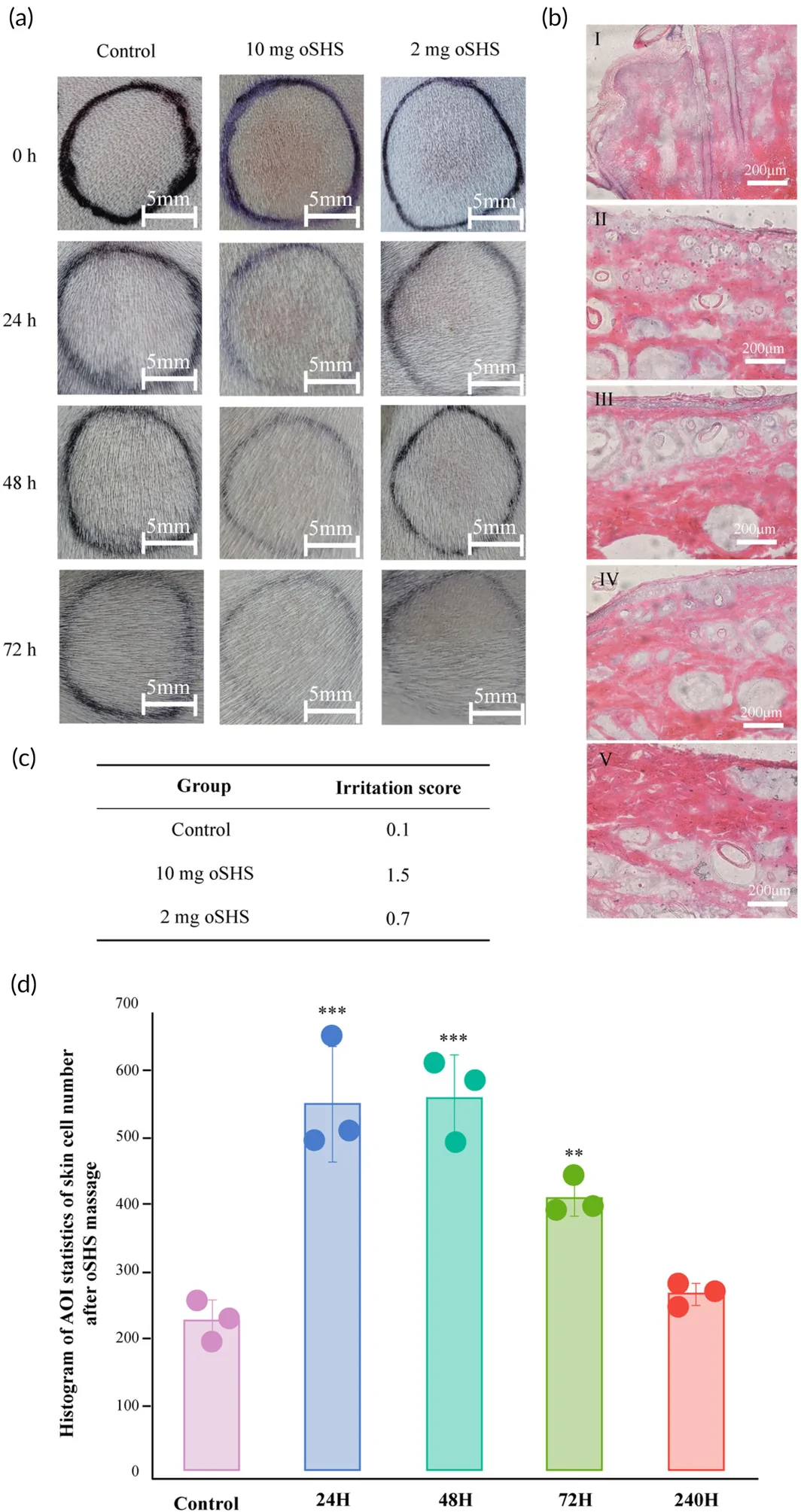
(a) Changes in the surface of guinea pigs skin over time after treatment with oSHS of different concentrations. (b) Pathological slice of guinea pig back skin treated with oSHS, I: Control, II: 2 mg oSHS 24 h, III: 2 mg oSHS 48 h, IV: 2 mg oSHS 72 h, V: 2 mg oSHS 240 h. (c) The skin irritation intensity scoring. (d) The number of skin cells in the AOI (area of interest, 450 μm × 150 μm) in dermis. The data value is the mean ± SD (*n* = 3), where **: *p*< 0.01, ***: *p*< 0.001.

## 4 DISCUSSION

oSHS differs mechanistically from our previously reported SHS‐based systems. In our earlier studies, SHS enhanced transdermal delivery mainly through the persistent microchannels created after skin puncture. This platform was subsequently extended by combining SHS with auxiliary strategies, such as flexible liposomes for high‐molecular‐weight hyaluronic acid and cationic flexible liposomes for siRNA delivery, or by surface engineering SHS with a mesoporous silica coating to enable drug loading and local release.^20^, ^22^, ^29^ By contrast, NaOH‐etched oSHS introduces a continuous internal hollow channel within the spicule, thereby extending the transport pathway from the nano‐scale interstitial spaces associated with SHS to a stable micron‐scale channel.

In the present study, effective delivery was demonstrated for molecules up to 150 kDa, whereas larger macromolecules and nucleic acids were not directly evaluated. However, our previous studies showed that SHS combined with flexible liposomes enabled delivery of 250 kDa hyaluronic acid into deep skin layers,^20^ and that SHS combined with cationic flexible liposomes significantly enhanced siRNA skin delivery.^22^ Given the larger transport pathway provided by oSHS, combining it with appropriate auxiliary systems may further improve delivery of larger biomacromolecules and nucleic acids. Importantly, oSHS retains the potential for further surface modification, offering additional flexibility for future optimization.

Under the passive transport conditions used in this study, the fitted permeability behavior was interpreted using an empirical microporous framework. In this model, the dominant $\text{logM}W^{-0.6}$ term resulted in an overall negative relationship between permeability and MW across the tested range, and this trend was retained in the sensitivity refit, indicating that the model remained consistent with diffusion‐related MW dependence. Additional details of the sensitivity analysis are provided in the Supplementary Note.

Nevertheless, several translational considerations warrant discussion. Silica is a recognized granulomatogenic material, and cutaneous implantation of silica‐containing particles may trigger a foreign‐body granulomatous reaction. Clinically, cutaneous silica granulomas may present as indurated papules or nodules months to years after exposure, reflecting the potentially delayed nature of this response.^30^ Mechanistic studies have suggested that silica can induce granulomatous inflammation through non‐allergic colloidal or foreign‐body pathways.^31^ Recent dermatological reports have also raised safety concerns regarding cosmetic use of marine‐derived silica spicules applied by rubbing.^32^ While our 10‐day observation was sufficient to evaluate acute skin irritation, it does not address the potential for rare, late‐onset granulomatous reactions that may develop months after silica exposure. Future studies with longer follow‐up and broader dermatopathological evaluation, including assessment for birefringent particulates under polarized light, would enable a more comprehensive evaluation of delayed granulomatous reactions.

In addition to the acute irritation findings, the biocompatibility of oSHS should also be considered in relation to the etching process. Although residual NaOH was not directly quantified in the present study, the neutral pH of the final suspension, together with the mild and reversible skin response observed in vivo, suggests that the post‐etching process did not cause obvious chemical irritation. In addition, a repeated‐application study showed no significant chronic inflammatory cell infiltration or structural damage in skin sections collected at day 28 (Figure S5), supporting the local cutaneous tolerability of oSHS under repeated‐use conditions. Histological examination of major organs also showed no obvious pathological abnormalities after repeated application (Figure S7), providing preliminary evidence against detectable toxicity under the tested conditions. Nevertheless, the present evaluation was based primarily on histological observation, and a more comprehensive assessment of long‐term immunological safety, including cytokine profiling, lymph node activation, and other sensitization‐related endpoints, will be necessary to further define the immunotoxicity profile of oSHS.

An additional consideration is the tissue fate of oSHS after topical application. In our system, deep skin delivery results from biomacromolecule diffusion through oSHS‐generated hollow channels rather than dermal deposition of the spicules themselves. Previous studies from our group showed progressive disappearance of SHS from the skin over time, supporting clearance through epidermal desquamation rather than permanent retention.^20^, ^21^, ^29^

Scalability and batch‐to‐batch consistency are important for clinical translation. In our previous work, SHS derived from *Haliclona* sp. exhibited highly consistent length and diameter distributions across independent production batches, with no statistically significant differences.^22^ Given that penetration efficiency is primarily governed by geometric parameters and structural integrity, such dimensional reproducibility is expected to minimize functional variability. Moreover, as a naturally derived biomaterial, oSHS may provide a cost‐effective alternative to synthetic MN arrays, which typically require complex and expensive microfabrication processes. Nonetheless, standardized production control and quality‐assurance criteria will be essential for large‐scale application. In addition, because the current evaluations were performed in animal models, differences in barrier structure and immune responsiveness between animal and human skin may affect translatability; therefore, validation in ex vivo human skin and future clinical studies will be necessary.

Future studies should optimize iontophoresis parameters such as current density, duration, and waveform to maximize synergy with oSHS while maintaining an acceptable safety profile and practical usability for patients. Expanding the molecular weight range beyond 150 kDa and exploring nucleic acid delivery will further define the scope and limitations of this platform. Overall, oSHS represents a promising strategy for the skin delivery of biomacromolecules, and continued safety evaluation and translational validation remain essential.

## 5 CONCLUSION

Hollow sponge spicules (oSHS) significantly enhance the skin penetration and deposition of biomacromolecules in a dose‐dependent manner and are acutely safe. When combined with iontophoresis, oSHS further improves the skin absorption of these molecules. Biomacromolecules delivered topically via oSHS are evenly distributed within the skin, whereas subcutaneous injection results in accumulation at the injection site. In vivo, oSHS successfully delivered OVA, eliciting immune responses similar to subcutaneous injection. Overall, oSHS presents a promising strategy for the skin delivery of biomacromolecules.

## AUTHOR CONTRIBUTIONS

**Dezhen Mou:** Writing – original draft; validation; investigation; software. **Mengnan Jia:** Data curation; writing – review and editing. **Xinye Xiao:** Methodology. **Yuhan Liu:** Validation. **Jun Chen:** Methodology. **Minghan Yang:** Writing – review and editing; data curation. **Ming Chen:** Writing – review and editing; writing – original draft; funding acquisition; project administration; resources; data curation; supervision.

## CONFLICT OF INTEREST STATEMENT

M.C. is a shareholder and scientific advisor to Marinesharp Therapeutics.

## Supporting information


**Supplementary Figure S1.** Comparison of in vitro percutaneous extraction of model molecules (FD40 kDa) using SHS, oSHS and oSHS+ITP.
**Supplementary Figure S2.** In vitro percutaneous delivery fluorescence distribution of FD40 kDa.
**Supplementary Figure S3.** Comparison of delivery effects of oSHS group and oSHS+ITP group on in vitro percutaneous delivery of model molecules (150 kDa fluorescent glucan).
**Supplementary Figure S4.** In vitro percutaneous delivery fluorescence distribution of FD150 kDa.
**Supplementary Figure S5.** Histological evaluation of mouse skin after repeated oSHS application.
**Supplementary Figure S6.** Distribution of FD150 K in the oSHS delivery area.
**Supplementary Figure S7.** Histopathological evaluation of major organs in mice from different groups.

## Data Availability

The data that support the findings of this study are available from the corresponding author upon reasonable request.
