# Supplementary material for: Enhanced skin delivery of biomacromolecules using hollow sponge spicules
Source: Bioeng Transl Med. 2026 May 19;11(5):e70149. doi: 10.1002/btm2.70149 (PMC13636246; doi:10.1002/btm2.70149)
Supplement: Supplementary file 1 [file BTM2-11-e70149-s001.docx]

**Supplementary Figures：**

**Supplementary Figure S1. Comparison of in vitro percutaneous extraction of model molecules (FD40 kDa) using SHS, oSHS and oSHS+ITP**

The data values are mean ± standard deviation (n = 3), *: *p*<0.05, ***: *p*<0.001

**Supplementary Figure S2. In vitro percutaneous delivery fluorescence distribution of FD40 kDa**

A: Untreated control group; B: SHS group; C: oSHS group; D: oSHS+ITP group

**Supplementary Figure S3. Comparison of delivery effects of oSHS group and oSHS+ITP group on in vitro percutaneous delivery of model molecules (150 kDa fluorescent glucan)**

The data values are mean ± standard deviation (n = 3), ***: *p*<0.001

**Supplementary Figure S4. In vitro percutaneous delivery fluorescence distribution of FD150 kDa**

A: Untreated control group; B: 2 mg oSHS group; C: 5 mg oSHS group; D: 10 mg oSHS group; E: 10 mg SHS group; F: Roller microneedle group; G: 10 mg oSHS+ITP group

**Supplementary Figure S5. Histological evaluation of mouse skin after repeated oSHS application.**

oSHS was applied topically once every three days for 28 days (9 applications total). Skin tissues were collected on day 28 and stained with H&E.

**Supplementary Figure S6. Distribution of FD150 K in the oSHS delivery area.**

Following oSHS application, skin samples were collected from three random sites within the treated area, and FD150K concentrations in the dermal layer were quantified.

**Supplementary** **Figure S7. Histopathological evaluation of major organs in mice from different groups.**

oSHS was applied topically once every three days for 28 days (9 applications total). Organs of heart, liver, spleen, lung, and kidney were collected on day 28 and stained with H&E.

**Supplementary Note：**

The original fitted permeability equation contains a small positive linear MW term (+0.0000289·MW). To test whether this term affects the main conclusion on molecular-weight dependence, we performed a sensitivity analysis by refitting the regression after removing the linear MW term.

Original regression (Line 431):

$\log k_{p}=19.107+0.0000289\mathrm{MW}-1.3501\log P+4.4579\log\mathrm{MW}^{-0.6}$ (R^2^=0.7663)

Sensitivity refit (without the linear MW term):

$\log k_{p} = 8.335 - 0.295\log P+ 1.228log {MW}^{-0.6}$ (R^2^=0.687)

Although removing the linear MW term modestly reduces the goodness-of-fit, the refitted model preserves the same diffusion-consistent MW dependence via the $MW^{-0.6}$ scaling (overall permeability decreases with increasing MW). This supports the interpretation that the small positive linear MW coefficient in the original regression is not required for the main conclusion and is more likely due to model instability in a limited dataset rather than a genuine non-diffusive transport mechanism.
